# And When I Die: Theory of Planned Behavior as Applied to Sperm Cryopreservation

**DOI:** 10.3390/healthcare9050554

**Published:** 2021-05-09

**Authors:** Limor Dina Gonen

**Affiliations:** Department of Economics and Business Administration, Ariel University, Ariel 40700, Israel; limorg@ariel.ac.il

**Keywords:** cryopreservation, Theory of Planned Behavior (TPB), Willingness to Pay (WTP), conjoint analysis (CA)

## Abstract

The present study investigates fertility intentions of men, aged 18–59, as expressed in willingness to cryopreserve sperm for future use in procreation. An economic stated-preference framework is combined with the Theory of Planned Behavior (TPB) to investigate which attributes are important in the decision to cryopreserve sperm, what is the Willingness to Pay (WTP) for cryopreservation, and which attributes influence it. A structured, two-part questionnaire was used, based on WTP and Conjoint analysis (CA) applied in tandem to elicit respondents’ preferences in evaluating utility. Findings show which attributes are important in the decision to cryopreserve sperm among them Risk of Infertility, Personal monthly income, Chance of pregnancy from frozen semen, Age and what are significant predictor variables for the WTP which are Personal monthly income, Importance of the risk of infertility, Initial registration fee to sperm bank and cryopreservation, and Degree of religious observance. The findings further demonstrate that respondents value sperm cryopreservation and have a positive WTP for it as it seems to contribute to improving well-being. As a result of these findings, governments should consider state funding for cryopreservation as part of national health policy.

## 1. Introduction

“And when I die, and when I’m dead

Dead and gone

There’ll be

One child born, in our world

To carry on...”

[Blood, Sweat, and Tears]

### 1.1. The Theoretical Framework

This study seeks to investigate cryopreservation intentions using combination of the Theory of Planned Behavior (TPB) with the economic stated preference framework. Sperm cryopreservation intentions, as manifested in the willingness to cryopreserve sperm for future use in procreation, are expressed in the Willingness to Pay (WTP) for cryopreservation. The TPB [1,2,3], has been employed for underlining the link between intentions and behavior at the micro and macro level. In the micro context, fertility behavior has been conceptualized as the result of a decision-making process which weighs the costs and benefits of possible actions. Sperm cryopreservation is a fertility behavior that indicates intention and can be examined if actually implemented, while considering personal characteristics and factors. These include subjective norms (the individual’s perception of psychological support or social pressure), perceived behavioral control (how the individual perceives the relative ease or difficulty of performing the behavior or achieving the goal), all impacting intention and behavior [4]. Through questionnaires, factors such as beliefs, attitudes, social norms (e.g., norms about childbearing age, etc.) as well as broader national or cultural values and economic and political context are examined. The TPB also incorporates the macro context [5,6] that can be evaluated through an economic perspective, reflected in the conclusions regarding the financing or subsidy of sperm cryopreservation by the public authorities.

### 1.2. The Aims of the Study

Human sperm cryopreservation has become widely accepted all over the world. The present study investigates fertility intentions of men, as expressed in the willingness to cryopreserve sperm for future use in procreation. The innovative combination of the TPB with the economic stated preference framework enables an investigation of issues related to this technique by applying a technique known as Conjoint analysis (CA) that can take into account respondents’ preferences in the evaluation of utility.

The study aims to investigate which attributes are important in the decision to cryopreserve sperm; which attributes influence the WTP for cryopreservation; what is the WTP for cryopreservation, and as a corollary, whether the government should fund cryopreservation. The study’s findings demonstrate that respondents value sperm cryopreservation and have a positive WTP for it, and are willing to pay, on average, an annual sum that is higher than the average annual cost of cryopreservation. Sperm cryopreservation appears to deliver substantial benefits and contribute to improving well-being.

### 1.3. Sperm Cryopreservation

Human sperm cryopreservation is an option that may enable the fulfillment of what is considered to be one of the most important goals in life—having children. In case of serious illness, premature death, reproduction capacity that decreases with increases age, increase risk of heritable disease-causing mutations because of late parenthood and risk for fertility in certain professions having the possibility of cryopreservation may provide consolation through genetic continuity. The importance of biological parenthood, i.e., genetic relatedness, has deep cultural and biological roots [7,8]. The desire for biological parenting and the importance attached to it overrides other possibilities such as adoption or the use of donor gametes as alternatives to reproductive technologies aimed at the fulfilment of ‘biological parenthood’ [9,10,11].

Sperm cryopreservation has become widely accepted all over the world [12]. It has been proven to be a very successful method and even become routine procedure to preserve the possibility of male procreation [12,13,14].

The last two decades have seen a steady increase in male fertility preservation. Important progress has been made in treating various types of cancer and other serious chronic medical conditions; consequently, patient survivorship has risen and with it, a demand for preserving the ability to produce offspring. There has been a similar increase in nonmedically indicated fertility preservation, which has given rise to the proliferation of social sperm banks, gender dysphoria prior to gender-affirmation surgery, and sperm preservation for posthumous reproduction. The optimal technique used for male fertility preservation is sperm cryopreservation, and while auto-transplantation of testicular tissue and spermatogonial stem cell is still regarded as experimental, it offers an encouraging alternative for prepubescent patients [14].

#### 1.3.1. Oncological Applications

Fertility preservation for young patients has become an issue of paramount importance, as medical progress has brought about a situation where most children (0–19 years), adolescents, young adults and adults (those up to 45 years of age) with reproductive capability who are diagnosed with cancer will survive the disease and in the future wish to father children [13,15,16,17]. Given the long-term effects of cancer treatment on fertility, the preservation of sperm is a promising option [18,19,20,21,22,23,24]. The cryopreservation of sperm before beginning gonadotoxic therapy is a central means of ensuring this reproductive option [25]. Sperm is most usually obtained by means of masturbation, although when not feasible (e.g., in pre-pubertal boys who do not produce mature sperm cells and in post- pubertal males who cannot produce a sperm sample) [26], other techniques are employed, both surgical and non-surgical, including electroejaculation, electrical stimulation, microsurgical epididymal sperm aspiration, and testicular sperm extraction (TESE). The cryopreservation of testicular tissue is being investigated as an option when other techniques are not possible [27]. Parents are required to give consent for decisions concerning fertility preservation for children under the age of 18. Parents must undertake the burden of responsibility, then, for their young children, and at times, for adolescents and young adults who are too stressed to make fertility decisions alone and trust their parents to initiate procedures [28,29] and help in crucial decision making. Factors to be considered are potential health risks arising from the procedures, delays in cancer treatments, raising false hopes in patients and their parents about the chances of surviving cancer and the successful implementation of fertility preservation procedures. There is also a potential for misinterpretation of the risks as well as overly optimistic hopes of fertility preservation success [30,31]. Fertility preservation is generally a new field of information for parents, while patients’ involvement in the decision-making process varies from patient to patient [24,32]. Generally each fertility preservation option has its own inherent benefits and risks that must be weighed together with personal values [33]. The ultimate decision is not a simple one to make, considering the ethically complex nature of the situation.

#### 1.3.2. Non-Oncologic Medical Conditions

Sperm cryopreservation is indicated for men with non-cancer-related medical conditions including major trauma injury (such as spinal cord injuries) and chronic autoimmune disease [34,35,36]. Patients who elect to undergo a vasectomy should be apprised of the option of either sperm cryopreservation before the procedure or sperm extraction from the ducts during surgery, since vasectomy is intended to be permanent. Although candidates for vasectomy are asked if they are certain, they will not want the capability to biologically father children in the future, some eventually request a reversal [37]. HIV-positive individuals, who can now expect a longer lifespan, have also become interested in preserving their reproductive capabilities [38,39]. Sperm cryopreservation is an important option for them because HIV infection could damage sperm quality and fertility capacity, especially in advanced stages of the disease.

#### 1.3.3. Posthumous Sperm Retrieval

In posthumous sperm retrieval, the gametes of a deceased individual are preserved for the aim of producing a genetic offspring in the future. This medical option gives rise to a range of ethical, moral and legal dilemmas for all parties directly concerned: patients, their families, and clinicians, as well as for society at large [40,41].

#### 1.3.4. Men Undergoing Gender Reassignment

In the case of individuals who seek to undergo gender reassignment (gender dysphoria), the purpose of fertility preservation is to maintain their capability to sire biological offspring [14,42]. Fertility preservation is important in the health management of adolescents and young adults who contemplate medical and/or surgical treatment that could impact their future fertility [43,44,45]. For this reason, the potential risk to fertility should always be considered [43,46,47] and ways explored to preserve fertility prior to undergoing any medical and/or surgical management. Furthermore, patients and their families should be helped to understand the potential effects of medications and/or surgical procedure and become knowledgeable about assisted reproductive technologies [48].

#### 1.3.5. Non-Medical Indications for Fertility Preservations

Because people are marrying later and childbearing is delayed, ‘social sperm cryopreservation’ is increasing among men concerned by the mounting evidence that postponing fatherhood may increase the risk of heritable disease due to reduced quality of sperm. With age, reproductive capacity declines as does the sperm count, as seen during the last four decades [49]. Advanced paternal age (APA) at 40 years and above [50] reduces fertility and is associated with a decrease in both natural fertility and the success rates of ART treatments. Also seen to be affected by APA are pregnancy outcomes [51] and neonatal risks. There is increased evidence that the spermatozoon of older men increases the risk of a variety of pregnancy complications [50], including certain childhood cancers [52], autism [53], birth defects [54] and some psychiatric disorders [55]. All of these indications of adverse outcomes for fertility and offspring should encourage clinicians to counsel men about the associated potential risks associated with delaying fatherhood. Sperm freezing has also become a commercially available option for preserving the reproductive capacity of men in jobs or sports that may risk their fertility (e.g., working in toxic environments, playing hockey, football). Men serving in the military or security services who encounter life-threatening situations may consider the option of cryopreservation before they enter a conflict arena [56,57,58].

### 1.4. Barriers to Effective Sperm Cryopreservation

Since the 1950s, it has been possible to cryopreserve sperm successfully as a means to preserving male fertility. Yet despite this long history, as well as the fact the process can be carried out with relative ease and speed, relatively few men actually avail themselves of this option before beginning cancer treatments and even fewer do so for other, non-medical reasons [59]. The explanation for this may be insufficient information- clear and unambiguous information is important [60]; expense—if not covered by health insurance or state-funded [14], and some men are apparently reluctant to submit to semen analysis [61]. This indicates that there are barriers to effective sperm cryopreservation, which may be connected to the subjective norm and perceived behavioral control.

#### Subjective Norm and Perceived Behavioral Control

A subjective norm is the individual’s perception of the psychological support or social pressure which people in their close social circle exert on them to perform or not perform a certain behavior or achieve a goal; it is ‘subjective’ in the sense that it does not necessarily correspond accurately to the actual opinions of other people or the accepted societal norms. The more favorable the attitude and the subjective norm regarding the decision to cryopreserve sperm the more likely it is that an individual will formulate the intention of cryopreserving sperm. Perceived behavioral control refers to how the individual perceives the relative ease or difficulty of performing the behavior or achieving the goal; here, too, the key word is the individual’s perceptions regarding the situation. The greater the perceived control, the more likely it is that an individual will formulate the intention of cryopreserving sperm. Sperm cryopreservation intentions are also certainly likely to be affected by societal norms. Since individuals do not live in isolation but are part of social environments [62,63].

### 1.5. The Theory of Planned Behavior

The TPB [1,2,3] has been employed as a framework for assessing intentions which are strong predictors of behavior. The intentions were assessed by evaluating attitudes, beliefs and preferences through questionnaires.

Based on the TPB, it can be concluded that sperm cryopreservation intention stems from and is based on the following factors [6,63,64]:

(1) The individual: personality; emotions; intelligence; values; general attitude etc.
(2) Sociodemographic characteristics: age; gender; income; education; religion; family status
(3) Societal factors: social norms; culture; political context
(4) Behavioral beliefs—beliefs regarding the consequences of fertility decisions
(5) Normative beliefs—beliefs about societal support for fertility decisions, including beliefs concerning social support for cryopreservation—perceived societal norms or social pressure relating to cryopreservation.
(6) Control beliefs—beliefs about factors that enable or impede cryopreservation. i.e., factors that may potentially influence the individual’s ability to freeze sperm for later use (low sperm count, illness, expense, etc.).

To understand the attitudes towards cryopreservation, it is essential to obtain accurate information about the above factors that play a key role in an individual’s decision to opt for cryopreservation [4].

The questionnaires in the present paper were designed to examine intentions of sperm cryopreservation that are affected and influenced by subjective norms, perceived behavioral control, and social norms.

The questionnaires include questions regarding:

- The emotional reactions toward possible infertility—Decisions made about sperm cryopreservation might be based on values, general attitude, fear, risk aversion, limited resources, and social norms.
- Stage in life, i.e., socio-demographic status—Decisions are influenced by the stage in life of the respondents, such as age, education, income, degree of religiosity, and family status.
- Control factors, including costs—Decisions are influenced by external factors such as costs.
- Valuation utility—Decisions are influenced by an evaluation of the private benefit and the social benefit.

### 1.6. Social Benefit and Public Funding

The discussion of social benefit is significant when considering public funding.

The advances in sperm cryopreservation technique have led to the controversial dilemma of who should fund elective sperm cryopreservation.

In many Western countries today, sperm freezing for non-medical purposes is only available for men who have the means to pay for the procedure and the storage fees. Shanner [65] observes that although the right to reproduce is largely recognized as a liberty-right, it is not generally regarded as a claim-right.

The right to procreate is surrounded by ambiguity as reflected in its content and scope, the values on which such a right is based, and the legal and social frameworks guaranteeing this right, despite the fact that the right to procreate has been recognized in case law. Such ambiguity compounds the difficulties associated usually with the discourse on rights, and in particular, the law’s intervening in bioethical issues, through the language of rights [66,67]. Pearson [68] shows that there is controversy over whether the right to procreate is positive, such that individuals can require others to support their reproduction process, or if it is negative, so that it only mandates that others do not interfere in their reproductive choices. The argument for a positive right to procreate includes a claim-right to receiving help from the state and its agencies which are obligated to provide such assistance. This would require medical institutions to provide artificial insemination facilities to all applicants without their fulfilling specific criteria. Moreover, the procreative right entails immunities, meaning that no one, including the state, can reduce the procreative claim rights, liberties, and powers of the potential procreators. Robertson [69], a firm proponent of procreative liberty, asserts that “reproductive rights are rights against the state limiting or restricting an individual’s reproductive choices or efforts to obtain reproductive services from a willing provider.” They are not rights to have the state provide the services or resources needed ([69], p. 20).

When it comes to non-medical ‘cryopreservation’, i.e., social sperm cryopreservation, this means that while men may be free to cryopreserve their sperm if they so desire, they cannot make claims on society to fund their efforts [70].

However, since medical and non-medical fertility preservation is on the rise and becoming more common, the question is asked about whether ‘non-medical freezing’ should be covered by public healthcare (or required by insurance coverage) meaning that elective sperm cryopreservation should also be funded by the society.

Storage and fertility treatments vary in cost across countries. Whereas some countries cover total financial costs through the public health system, other countries have only private health services and thus the financial status of the patient ultimately decides their options [14,71,72,73,74].

## 2. Methods

### 2.1. The Logic of the Empirical Model

Insights into how patients value different aspects of health care procedures are essential for designing and assessing programs. Policy decisions that relate to clinical, licensing, and reimbursement considerations can be improved by incorporating values that most closely reflect the preferences of the patients. Adapting health care policy to patient preferences by improving satisfaction with clinical treatments and public health programs could do much to enhance the effectiveness of health care procedures [75,76,77].

There are two distinct approaches to the measurement of preference as defined by economists: revealed and stated [78], both based on the same theoretical foundation. Revealed preferences are derived from actual observed market activities and are identified by complicated econometric methods employed by researchers. Stated preferences are obtained from surveys which allow researchers to control how preferences are elicited.

Stated-preference methods are divided into two categories:

- Methods using direct elicitation of monetary values of an intervention (including contingent valuation or WTP and Willingness-to-Accept methods) [78,79]—designed to evaluate the demand for a single product, or
- Methods using rating, ranking, or choice designs (either individually or in combination) to quantify preferences for different properties of an intervention (usually known as CA, discrete-choice experiments, or stated-choice methods)—designed to investigate trade-offs between the properties of a product and its impact on preference.

### 2.2. WTP in Health Care Studies

The WTP method is typically utilized to assign a monetary value to health benefits relating to a specific healthcare intervention, for the purpose of eliciting the patients’ values and preferences as well as the general public’s attitude [80] toward various interventions and to enable an overall assessment of benefits as perceived by the respondents [81,82,83,84,85,86].

### 2.3. Conjoint Analysis in Health Care Studies

Health care studies have seen a surge in the application of CA studies [87,88,89]; for a detailed literature review, see Clark et al. [90] and De Bekker-Grob et al. [91]. CA is a method where part-worth values for individual attributes are derived from a total score for a good or service comprised of two or more attributes and is based on ranking a set of values [92,93,94,95]. Such a method is especially appropriate for quantifying preferences for goods and services which are not traded on the market or where regulatory mechanisms or legal constraints limit market choices, for example, in the case of health care services and products [96]. CA has been used effectively to measure preferences for a wide variety of health applications [77,89,97,98,99,100,101], although it has potential value in assessments apart from health care interventions. It is also being used increasingly to understand preferences for health-related quality of life (health state) and as a means to evaluate the outcomes of various health states reported by patients [102,103]. CA has also aroused the interest of licensing authorities as a method of evaluating patients’ readiness to place themselves at risk in undergoing innovative treatments which promise to be more effective [104]. CA offers a mechanism for facilitating decision making both for patients to participate in [105,106] and for shared decision making [107] as well as to understand clinical decision making [108] and how the various parties at interest valuate healthcare outcomes [109].

In addition to valuating the relative importance of one or more attributes of a good or service, CA may be used to assess how individuals trade off between these attributes. This refers to the rate at which the user is willing to exchange one unit of an attribute for another [110].

In a CA study, hypothetical scenarios are presented with attributes of a good or service considered to be of varying levels of importance. Respondents are asked to rank the services, rate them, or choose paired attributes. While individuals commonly make choices relating to exchange and substitution on a daily basis, they seldom are called upon to rank and rate attributes in everyday decision making. The contribution of this paper is in developing and applying the pairwise ‘choice’ approach employed in the decision-making process, which is used as a comparison of two indirect utility (or benefit or satisfaction) functions. For the study, the respondent is asked to make a series of pairwise choices. For each comparison, the respondent chooses (or prefers) the alternative that leads to the higher level of utility.

### 2.4. Study Design and Methods

Two techniques were applied in tandem to elicit preferences: WTP and CA WTP theory predicates that the amount of money a person is willing to give for a benefit in healthcare is an indicator of how much that person values the benefit [111]. CA helps in determining what value individuals assign to different components of a particular health product or service [96]. By analyzing how respondents specify their preferences for various characteristics of the service or good, the utility, i.e., implied value, of the specific components of the health treatment can be determined. The analysis of CA in the present paper is based on the paper of Ryan [110].

For this study a structured, two-part (WTP and CA) questionnaire was designed:

(1) A questionnaire was distributed to ask men directly about their WTP for sperm cryopreservation. First, the participants were given a description of the product to be evaluated—sperm cryopreservation, and then they were asked about their WTP for the product. Their maximum WTP was determined from a combination of double-bounded questions (closed-ended) (see Appendix A, Table A1).
(2) For CA, respondents were given hypothetical scenarios involving different levels of attributes which were identified as being important attributes in cryopreservation, and they were asked to make pairwise choices. The preliminary set of attributes and their levels were determined by a literature review. Table A2 in Appendix A summarizes the attributes and levels included in the CA study.

Respondents were shown a set of 10 scenarios, consisting of Option A and Option B. Option A consisted of fixed attributes, while Option B was different in each scenario, so that the CA questions consisted of 10 pairwise choices (see Appendix A, Table A3). Table A4 in Appendix A presents an example of one of the pairwise choices.

A diagram presenting the methodology according to the objective which are pursued, and the methodology used is presented in Appendix B (see Appendix B, Figure A2).

### 2.5. Experimental Design & Methods

#### 2.5.1. Data Gathering

Data was gathered by means of surveys conducted among men from the general public. The study respondents were drawn from a pool of participants recruited through a survey company, who participated of their own free will and did not receive any monetary compensation for their participation. Only the survey company had access to data on the participants. Each participant was given a personal code so that the personal information was not known to the researcher in charge of the study.

The participants, who were all 18 years of age and over, were given a page describing the goals of the study, guaranteeing anonymity, and explaining the possibility of terminating their participation at any point in time. Participants were asked to sign an informed consent form before answering the questionnaire.

Three data-gathering stages were used to construct the survey and carry it out:

Primary Stage: In the primary stage, items to be included in the research questionnaires were identified, using in-depth interviews with five fertility experts and ten potential candidates for semen cryopreservation. The questionnaires were initially constructed on the basis of content analysis of interview results.

Pilot Study: After completing the first version of the questionnaires (based on the primary stage findings), a pilot study was conducted, with 50 participants. The pilot study aimed at assessing the difficulty and clarity of the questionnaire and the respondents’ willingness to respond to the various items it comprised. This pilot study, which included face–to-face interviews conducted by the researcher, provided the participants with detailed information about cryopreservation and enabled the presentation of relevant information in a supervised manner as it gathered responses to the different factors.

Main Survey: Based on the findings of the pilot study, the research questionnaires were revised and adapted, and then final versions were constructed for the survey population. The population sample consisted of Israeli Jewish men aged 18–59, from four major urban centers (Four large cities in four major population regions in Israel: Tel Aviv, Jerusalem, Haifa, and Beer Sheba). First, the survey company made contact by telephone, then questionnaires via Google Docs were send to respondents who agreed to participate in the study. Every respondent confirmed his participation by electronically signing an informed consent form.

Out of 750 questionnaires distributed, 597 valid questionnaires were filled out (79% response rate) by the participants. The final sample, after eliminating 98 respondents (because of inconsistency i.e., according to internal (theoretical) consistency tested through the CA technique. See Section 3 Methodological issues addressed) consisted of 499 men.

#### 2.5.2. Ethical Approval

Anonymous, self-administered questionnaires were filled out without experimental interventions. In the cover letter attached to the questionnaire, the subjects were informed that data collection and analysis would be kept fully anonymous, and their personal information would be fully protected; all answers would be kept confidential, processed statistically, and used for scientific research only. The subjects were free to decide whether or not to participate. Each participant provided signed informed consent to participate in the study.

## 3. Data Analysis

The SPSS version 26 was used to analyze the data.

A statistical descriptive analysis was performed to investigate the social and demographic characteristics of the respondents who took part in the study. Table 1 summarizes the social and demographic characteristics of the research sample.

Respondents’ Age, Education, Personal Income, Degree of Religious Observance, Marital Status were included as demographic variables in the model.

Age was used as a continuous variable, Marital status was used as a dichotomous variable, dichotomously coded as married versus not married.

Education was considered a categorical variable with four categories:

Elementary School—1st grade—9th grade, age range 6–15.

High School full education—10th grade—12th grade, age range 16–18.

High School and Post High School partial education:

High School—10th grade—12th grade, age range 16–18—partial education.

Academic degree—college, university—partial education.

Academic degree full education—college, university.

Personal Income was considered a categorical variable with five categories (Since the study was conducted in Israel, the monetary values were measured in Israeli New Shekels. I converted into dollars according to the dollar exchange rate on 23 February 2021, whereby, 1 ILS = 0.3063USD. The resulting dollar values were rounded according to mathematical rules for rounding numbers)

<$1226

$1226–$2145

$2145–$3065

$3065–$3984

$3984+

Degree of Religious Observance (In Israel, religious observance is a demographic factor that is used widely as a way for people to define themselves regarding their religious beliefs and practices. This is relevant when dealing with matters of reproduction, which are regulated and circumscribed by religious law and doctrine) was considered a categorical variable with three categories:

Degree of Religious Observance: Religious (The term ‘religious’ refers to Jews who follow the traditional Jewish religion)—varieties of Orthodox.

Secular (The term ‘secular’ is not strictly defined, and it can mean either “not religious” or “convinced atheists”)—Not religiously observant.

Traditional (The term ‘traditional’ covers a wide range of ideologies and levels of observance and is based on self-definition)—Observant of some fundamentals of religion.

A statistical descriptive analysis was also performed for the importance that respondents attribute to the following factors which are relevant to the decision to cryopreserve sperm. The importance respondents attribute to the risk of being infertile; The importance respondents attribute to the chances of initiating a pregnancy from sperm that was cryopreserved; The importance respondents attribute to the possibility of cryopreserving sperm for a chosen period of time; The importance respondents attribute to the initial registration fee and providing semen to the laboratory for cryopreservation (onetime fee); The importance respondents attribute to the annual price for cryopreservation and storage (must be paid annually). Table A5 and Table A6 in the Appendix A summarize the statistical descriptive and correlational analyses of the mentioned characteristics of the research sample.

Table A5 portrays that the most important factors relevant to the decision to cryopreserve sperm are (in decreasing order):

The importance respondents attribute to the risk of being infertile.

The importance respondents attribute to the chances of initiating a pregnancy from sperm that was cryopreserved.

The importance respondents attribute to the possibility of cryopreserving sperm for a chosen period of time.

The importance respondents attribute to the annual price for cryopreservation and storage (must be paid annually).

The importance respondents attribute to the initial registration fee and providing semen to the laboratory for cryopreservation (one-time fee).

**Figure A1:**
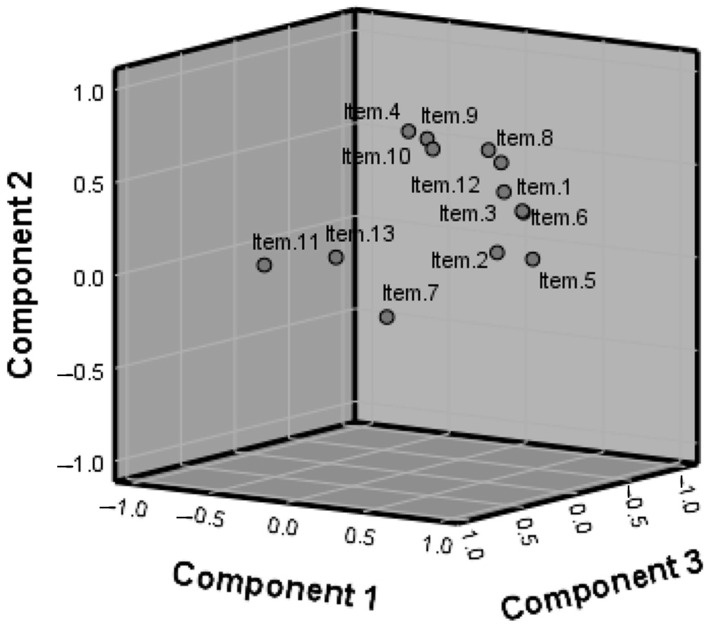
Component plot in rotated space, based on PCA and factor loadings.

**Figure A2:**
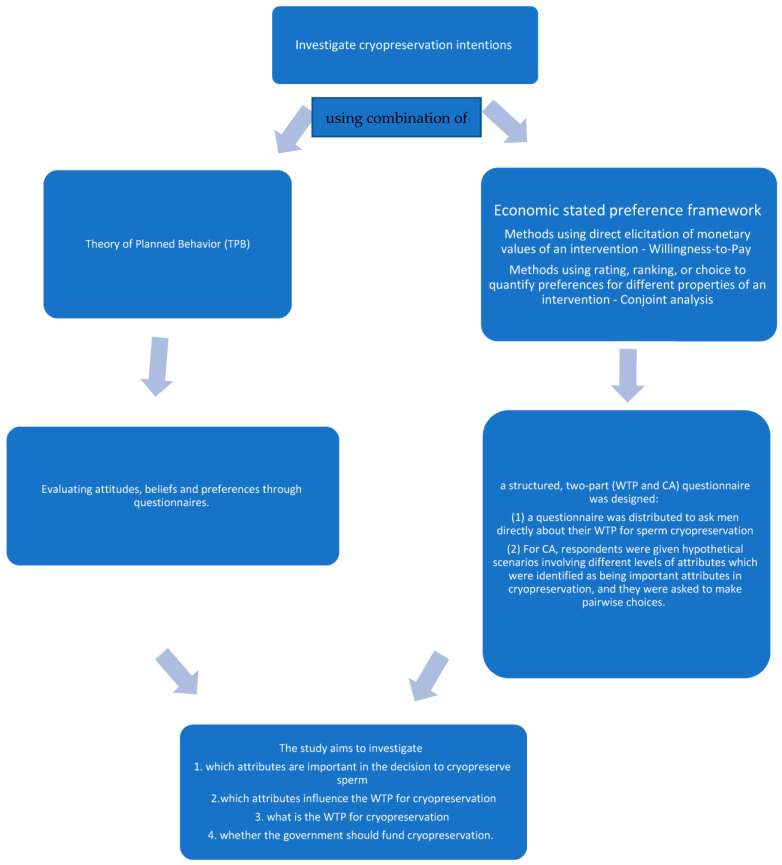
The methodology according to the objective which are pursued and the methodology used.

An exploratory factor analysis of the opinions pertaining to claims regarding cryopreservation was carried out using Principal Component Analysis, Rotation Method: Varimax with Kaiser Normalization. This analysis yielded three factors which cumulatively explained 61% of the variance. Eigen values are 3.52, 2.89, and 1.52 for the following factors, respectively: (a) Support 27.09% of the variance; (b) Continuation—explains 22.25% of the variance, (c) Justification—explains 11.69% of the variance; and. KMO (Kaiser-Meyer-Olkin) statistic was 0.882 which indicates a high adequacy of the data to the factor analysis. Table A7 in Appendix A summarizes the statistics of the opinions pertaining to claims regarding freezing cryopreservation. Table A8 in Appendix A present the Principal Component Analysis. In addition to the tabulate presentation of the Principal Component Analysis and factor loadings, Figure A1 in Appendix A, demonstrates the factors’ solution in a 3D graphical fashion. It is important to note, however, that factor 3 (Justification) had a low reliability coefficient, albeit excellent factor analysis indices (such as: loadings, KMO and eigenvalue). Hence, the factor was retained in further analyses. Table A9 in Appendix A displays the correlations among the factors construed by the factor analysis.

Table A8 and Table A9 in Appendix A indicate that each factor is a unique construct comprised of highly correlated items (i.e., high factor loading, above 0.55), and lack of multicollinearity among the factors themselves.

A Binary Logistic Regression was used to estimate the change in utility in moving from one scenario to the second scenario.

The regression function estimated is denoted by Equation (1).

∆V = β0 + β1 Agei + β2 Education Eli + β3 Education Hcpi + β4 Education Hcfi+ β5 Education ACpi + β6 Education ACfi + β7 Education pHci + β8 Personal Income Ai +β9 Personal Income Bi + β10 Personal Income Ci + β11 Personal Income Di + β12 Personal Income Ei + β13 Religious Observance Rei + β14 Religious Observance Tri + β15 Religious Observance Sei + β16 Marriedi + β17 Risk of infertiliti + β18 Chance of pregnancy from frozen semeni + β19 Semen frozen for period choosi + β20 Initial registration pricei + β21 Annual pricei + β22 Factor Supporti + β23 Factor Justificationi + β24 Factor Continuationi + ε(1)

CA was estimated in accordance with the function of the form:

$$\begin{array}{l} \Delta V\\ =\alpha_{1}\mathrm{risk}\;\mathrm{of}\;\mathrm{Infertility}+\alpha_{1}\mathrm{Chance}\;\mathrm{of}\;\mathrm{pregnancy}\;\mathrm{from}\;\mathrm{frozen}\;\mathrm{semen}\\ +\alpha_{3}\mathrm{Sperm}\;\mathrm{can}\;\mathrm{be}\;\mathrm{frozen}\;\mathrm{for}\;a\;\mathrm{period}\;\mathrm{of}\;\mathrm{my}\;\mathrm{choosing}\\ +\alpha_{4}\mathrm{The}\;\mathrm{initial}\;\mathrm{registration}\;\mathrm{price}\;+\alpha_{5}\mathrm{Annual}\;\mathrm{freezing}\;\mathrm{price}\\ +\alpha_{6}\mathrm{factor}\;\mathrm{support}+\alpha_{7}\mathrm{factor}\;\mathrm{justification}+\alpha_{8}\mathrm{factor}\;\mathrm{continuation}\\ +\alpha_{9}\mathrm{WTP}+\alpha_{10}\mathrm{trad}+\alpha_{11}\mathrm{religious}+\alpha_{12}\mathrm{age}+\alpha_{13}\mathrm{education}+e+u \end{array}$$

ΔV is the change in utility in moving from one scenario to the second scenario.

Risk of Infertility is the difference in the percentage of the chances of becoming infertile between one scenario and the second scenario. 

Chance of pregnancy from frozen semen is the difference in the Chance of pregnancy from frozen semen between one scenario and the second scenario.

Sperm can be frozen for a chosen period of time is the difference between the periods of time allowed for sperm cryopreservation between one scenario and the second scenario.

The initial registration price is the difference in the initial registration price between one scenario and the second scenario.

Annual freezing price is the difference in the registration freezing price between one scenario and the second scenario.

Factor support is the first explanatory factor.

Factor continuation is the second explanatory factor.

Factor justification is the third explanatory factor.

Occupation religion, age and education are the sociodemographic variables.

The unobservable error terms are represented by *e* and *u*, where *e* is the error term due to differences amongst observations and *u* is the error term due to differences among respondents.

### Methodological Issues Addressed

When using the CA technique, it is important to include an evaluation of whether individuals appear to understand the technique and relate to it seriously. This study tested for internal (theoretical) consistency and validity [110].

To check internal consistency, the rationality of the choices made was tested, i.e., if one scenario is clearly ‘better’ than another, respondents are expected to choose that scenario. In choice 7, the expectation is that all respondents would prefer the second scenario over the first. The assumption about respondents who answered inconsistently was either they did not understand the questionnaire, or they were not taking it seriously; these responses were dropped/omitted from the analysis. The premise of CA is that individuals have continuous preferences so that a deterioration in the level of one attribute is always compensated for by an improvement in another.

The regression analysis results were used to test the internal validity of CA, i.e., the extent to which the independent variables being tested are what led to the predicted results.

Given that higher levels of risk of infertility imply a problem, one would expect that the coefficient of the attribute Importance of the risk of infertility would be positive in the regression equation regarding the WTP for cryopreservation. One would expect the coefficient of the cost attribute to be negative regarding the WTP for cryopreservation. And one would expect that the coefficient of the attribute Personal monthly income would be positive in the regression regarding the WTP for cryopreservation.

**Figure A3:**
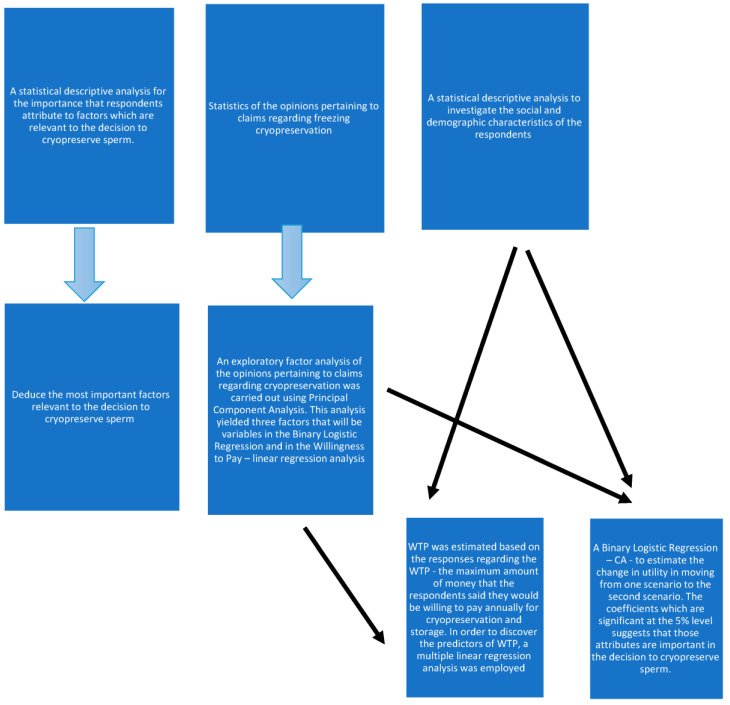
The list of the statistical analysis conducted.

A diagram with the list of the statistical analysis conducted presented in Appendix B (see Appendix B, Figure A3).

## 4. Results

### 4.1. Statistical Significance of the Attributes—CA—Logit Model

The coefficients which are significant at the 5% level are presented in Table 2 suggests that those attributes are important in the decision to cryopreserve sperm.

Table 2 indicates that the model has a good fit, and from all the predictors and interactions regressing on the probability that the respondents will choose 0 (scenario A) or 1 (scenario B) these are the statistically significant ones (ordered from the strongest effect on the outcome to the weakest effect, in absolute value):

Risk of Infertility (an increase in Risk of Infertility results in an increase in the probability to choose scenario B in 52%).

Personal monthly income (an increase in Personal monthly income results in an increase in the probability to choose scenario A in 68%).

Factor 3—Justification (an increase in the factor Justification results in an increase in the probability to choose scenario B in 30%).

Chance of pregnancy from frozen semen (an increase in Chance of pregnancy from frozen semen results in an increase in the probability to choose scenario B in 29%).

The interaction: Chance of pregnancy from frozen semen × income (the relationship between Chance of pregnancy from frozen semen and the probability to choose scenario A or B is conditioned/moderated by the Personal monthly income of the participant. As the Personal monthly income increases, the relationship between Chance of pregnancy from frozen semen and the probability to choose scenario A or B diminishes, indicating a strong importance to the Personal monthly income in this regard).

Importance of the risk of infertility (an increase in Importance of the risk of infertility results in an increase in the probability to choose scenario B in 23%).

The interaction: Risk of infertility × income (the relationship between risk of infertility and the probability to choose scenario A or B is conditioned/moderated by the Personal monthly income of the participant. As the Personal monthly income increases, the relationship between risk of infertility and the probability to choose scenario A or B diminishes, indicating a strong importance to the

Age (an increase in age results in an increase in the probability to choose scenario A in 84%).

Importance of Chance of pregnancy from frozen semen (an increase in Importance of Risk of Infertility results in an increase in the probability to choose scenario B in 14%).

WTP for cryopreservation (an increase in WTP results in an increase in the probability to choose scenario B in 9%).

### 4.2. Willingness to Pay—Linear Regression Analysis

WTP was estimated based on the responses regarding the WTP—the maximum amount of money that the respondents said they would be willing to pay annually for cryopreservation and storage.

The mean WTP for annual cryopreservation is $452 and the standard deviation (SD) is $839.

At the bivariate level, factor 1 (Support) is positively associated with WTP (r = 0.205, *p* < 0.001), and factor 2 (continuation) is also positively linked to WTP (r = 0.186, *p* < 0.001). However, factor 3 (Justification) has no significant relationship with WTP (r = 0.025, *p* > 0.05). the multivariate level of the analysis is presented in Table 3.

In order to discover the predictors of WTP, a multiple linear regression analysis was employed (method: Ordinary least squares). Prior to conducting the analysis, based on both—White’s test and Breusch-Pagan’s method for heteroscedasticity—the null hypothesis that homoscedasticity is present in the specific analysis was corroborated, thus minimizing the risk of biased regression estimators [111,112,113,114]. Additionally, Tolerance statistics indicate no risk of multicollinearity in the analysis, as shown in Table 3 (to avoid multicollinearity, a rule of thumb is: tolerance >0.40; [115,116].

Table 3 indicates that the model has a good fit. In certain disciplines, e.g., psychology, sociology and the social sciences, which explore human behavior, low R-squared values are common and anticipated. “Micro data on individuals, families, or households tend to have low R-squared because there is so much variation in individual behavior. Low R-squared do not necessarily mean that the model is poor” [117] p. 43. In the social sciences a relatively low R-squared in regression equations is not unusual. Ashenfelter and Kruege [118] based their conclusions on regression analyses which had R-squared of about 0.2 to about 0.3. Likewise, Levitt [119] reached significant conclusions from regression analyses which had R-squared of 0.06 to 0.37, while Kraai et al. [120] reports R-squared of 0.11. Effect size may also use adjusted R-squared as a measure [121]: small effect 0.0196, medium effect 0.1300 and large effect 0.2600). Savage [122] reports adjusted R-squared in the range 0.05 to 0.1. When using the contingent valuation method, findings of an adjusted R-squared in the range between 0.14 to 0.20 is considered normal and acceptable, see Spash et al. [123].

From all the predictors regressing on WTP, these are the statistically significant ones (ordered from the strongest effect on the outcome to the weakest effect, in absolute value):

Personal monthly income (positive association; the greater the Personal monthly income, the higher is the WTP).

Importance of the risk of infertility (positive association; the greater the Importance of the risk of infertility, the higher is the WTP).

Initial registration fee to sperm bank and cryopreservation (One-time payment) (negative association; the higher the Initial registration fee to sperm bank and cryopreservation [One-time payment], the lower is the WTP).

Degree of religious observance—Religious (negative association; the higher the religiosity level, the lower is the WTP).

Degree of religious observance—Traditional (negative association; the higher the religiosity level, the lower is the WTP).

## 5. Conclusions

Male fertility preservation has been increasing over the past two decades, both medical and non-medical. Fertility preservation prior to cancer treatments and in chronic medical conditions, as well as social sperm cryopreservation are becoming more common. “When timing and logistics are appropriate, sperm cryopreservation is considered the gold standard for fertility preservation” [14] p. 1.

The present study discusses social sperm cryopreservation among man. Social sperm cryopreservation is motivated by reproduction capacity that decreases with age [49], an increase in the risk of heritable disease-causing mutations because of postponing fatherhood [54], a risk to fertility in certain professions such as firefighters, policemen, and in advance of anticipated fertility damage during military service [64,124,125].

The literature regarding social sperm cryopreservation is sparse so this study may shed light on an area where research is needed. Moreover, the current study is pioneering and innovative since it examines social sperm cryopreservation from both the planned behavioral and the economic aspects.

The present paper analyzes cryopreservation intentions and the behavior they generate. The possibility an individual has today to decide upon sperm cryopreservation allows for the analysis of readiness and desire to cryopreserve sperm. TPB [1,2,3] has been used for exploring the link between beliefs and behavior. TPB is the most frequently used theoretical framework in the category of behavioral models [64]. Its underlying premise is the correlation between intention and behavior, with the former being a significant predictor of the latter. An examination of general attitudes, beliefs, and preferences is used in assessing intention. Within the TPB framework, cryopreservation is seen as the result of rationally taken decisions, based on the assessment of costs and benefits. The innovative combination of TPB with the economic stated preference framework enables the investigator to measure preferences using an economic approach. Two techniques were applied in tandem to elicit preferences: WTP and CA. WTP is the maximum amount of money a person would be willing to pay for cryopreservation, which is an indicator of the benefit from cryopreservation. CA helps in determining what value is assigned to different components of sperm cryopreservation [39], and thus what combination of attributes most influences the respondent’s choice or decision. By analyzing how respondents specify their preferences for various characteristics of sperm cryopreservation, the utility of the specific components can be determined.

One main question raised is which attributes influence an individual’s cryopreservation intentions, or more precisely, what would motivate a man to decide to cryopreserve sperm (i.e., what are the attributes that are important for making the decision to cryopreserve sperm). The study findings are that the attributes which influence the decision to cryopreserve sperm are:

Risk of Infertility, Personal monthly income, Chance of initiating Pregnancy from frozen semen, Importance of the risk of infertility, Age, WTP for cryopreservation, Factor 3—Justification (An exploratory factor analysis of the opinions pertaining to claims regarding the justification for cryopreservation), the interaction: Chance of pregnancy from frozen semen × income, and the interaction: Risk of infertility × income, both indicating a strong importance to the Personal monthly income.

Another question related to the predictors of WTP. According to this study the attributes that influence the WTP for cryopreservation are: Personal monthly income (positive association; the greater the Personal monthly income, the higher is the WTP, as it was theoretically anticipated and predicted [110]. Importance of the risk of infertility (positive association; the greater the Importance of the risk of infertility, the higher is the WTP), Initial registration fee to sperm bank and cryopreservation (One-time payment) (negative association; the higher the Initial registration fee to sperm bank and cryopreservation [One-time payment], as it was theoretically anticipated and predicted [110], the lower is the WTP), Degree of religious observance—Religious (negative association; the higher the religiosity level, the lower is the WTP), Degree of religious observance—Traditional (negative association; the higher the religiosity level, the lower is the WTP). Regarding religiosity and sperm cryopreservation the findings are interesting since for many people, moral and ethical decisions are based on the individual’s religious faith and beliefs. Cryopreservation, like many other relatively recent technologies, falls within the area where the major religions have not yet formulated guidelines or are still studying these issues in order determine acceptable guidelines. And while most religions have fundamental doctrines and worldviews that are constantly being applied to the rapid developments in medical and biological technologies, even major religions are not monolithic, divided as they are into geographical and cultural subgroups, with each putting emphases on their different ethical values and religious standards [126,127,128,129].

The state authority’s considerations were examined regarding funding cryopreservation by means of the national medical budget with its limited resources. The study’s findings demonstrate that respondents value sperm cryopreservation and have a positive WTP for it. The respondents are willing to pay, on average, an annual sum of approximately $452, an amount higher than the average cost of annual cryopreservation which is, on average, $185. Sperm cryopreservation appears to deliver substantial benefits and contribute to improving well-being. According to the economic literature, the funding of medical interventions should extend to the point where the costs of the medical intervention equal the social benefit from it. Based on the findings of this study, governments should consider funding annual cryopreservation. These results are potentially very useful to policy makers, especially since although sperm cryopreservation is the most cost-effective strategy for fertility preservation [128,129], it is underutilized [128,130,131,132]. Our results emphasize the need for decisive changes in public health policy.

Evidence has been presented that, within the context of cryopreservation, men are concerned about the possibility of infertility. This conclusion is not as obvious or intuitive as it seems. Studies regarding men’s views on reproductive masculinity [133] discussed the concept that men are less vulnerable to reproductive harm than women and consider themselves less responsible than women are for health problems in their offspring [133], In addition, even when men accept the existence of age-related fertility decline, they do not see it as related to their own personal lives [134]. An interesting finding from the CA analysis regarding risk is that respondents with a higher income had a lower marginal valuation of risk of infertility. Similar findings were presented in the literature regarding risks. Men tend to judge health risks as being lower than women do [135,136,137]. Finucane [84] found that risks tend to be judged as lower by men who had specific socioeconomic characteristics, including a higher income. Finucane [138] termed this phenomenon the ‘white male’ effect which later Palmer [139] called the ‘low risk’ effect.

CA has the potential to become an effective tool for assessing benefits in health economics. The current study had a relatively high response rate, as well as high levels of internal consistency and internal validity with results which are in line with findings from other studies [96,140,141,142,143,144]. CA was shown to be an effective technique for explaining the trade-off between attributes and also to be internally consistent and theoretically valid; it is also useful in estimating WTP indirectly.

Individuals are willing to trade off changes in the probability of reduction in the risk of infertility for other attributes. The ratio of the coefficients shows how much of one attribute an individual would be willing to give up for getting more of another attribute. The risk of infertility is more important than an approximately 2% increase in the chance of pregnancy from frozen semen. In other words, an individual would be willing to have a reduction (0.52/0.29 = 1.8%) in the chance of initiating pregnancy from frozen semen rather than to have an increase in the risk of infertility.

This paper presents highly novel and as yet unpublished data offering behavioral and economic insights into men’s perceptions of sperm cryopreservation and provides valuable insights for development of male reproductive health policy.

## Figures and Tables

**Table 1:** Social and demographic characteristics.

Variables	Males *N* = 499 %
Age	
22–18	6.6
27–23	45.2
32–28	20.3
37–33	9.6
42–38	7.2
43–47	5.9
48–59	5.2
Degree of Religious Observance	
Religious—Varieties of Orthodox	28.9
Secular—Not religiously observant	32.5
Traditional—Observant of some of the religious tradition	38.7
Education	
Elementary School	2.4
High School full education	22.4
High School and Post High School partial education	38.5
Academic degree Full education	36.7
Personal monthly income $	
<$1226	25.7
$1226–$2145	30.9
$2145–$3065	22.4
$3065–$3984	12.2
$3984+	8.8
Family Status	
Unmarried	40.9
Married	59.1

**Table 2:** CA—The Logit Model.

Variable	Exp(B)	SE	Wald’s Chi-Square	Pr > |t|
Intercept	0.15	1.26	1.55	0.00
Age	0.84	0.09	0.07	0.04
Education	0.73	3.03	3.00	0.80
Personal monthly income	0.68	0.30	0.24	0.01
Degree of religious observance—Traditional (The term ‘traditional’ covers a wide range of ideologies and levels of observance and is based on self-definition)	1.02	1.93	2.07	0.16
Degree of religious observance—Religious (The term ‘religious’ refers to Jews who follow the traditional Jewish religion)	2.50	0.02	0.01	0.29
Family Status—Married	2.34	3.37	11.18	0.09
Factor 1—Support	3.07	0.30	3.44	0.40
Factor 2—Continuation	4.53	0.22	3.25	0.32
Factor 3—Justification	1.30	1.20	5.90	0.03
Risk of Infertility	1.52	2.01	2.55	0.00
Chance of pregnancy from frozen semen	1.29	2.22	9.93	0.00
Option of sperm cryopreservation for chosen period of time	0.74	2.21	2.57	0.66
Price of initial registration to sperm bank and cryopreservation (one-time payment)	0.00	0.69	0.74	0.86
Annual fee for cryopreservation and storage (must be paid every year)	0.24	2.83	3.08	0.19
Willingness to Pay (WTP) for cryopreservation	1.09	0.21	0.19	0.04
Importance of the risk of infertility	1.23	0.09	0.12	0.00
Importance of Chance of pregnancy from frozen semen	1.14	7.07	7.16	0.01
Importance of the option of sperm cryopreservation for chosen period of time	0.82	0.76	1.82	0.18
Importance of registration fee- Initial registration to sperm bank for cryopreservation and storage (One-time payment)	0.98	3.58	3.66	0.28
Importance of annual fee to sperm bank for cryopreservation and storage	1.12	0.04	0.03	0.06
Risk of infertility × income	0.81	3.72	3.88	0.00
Chance of pregnancy from frozen semen × income	0.73	0.04	0.05	0.00
Option of sperm cryopreservation for chosen period of time × income	0.67	0.84	0.95	0.12
Initial registration fee to sperm bank and cryopreservation (One-time payment) × income	1.02	0.78	0.07	0.40
Annual fee for cryopreservation and storage (must be paid every year) × income	2.50	0.37	3.00	0.62
Chi-square fit test	48.45			
Pr > Chi-square	0.002			
Nagelkerke’s pseudo R-squared	0.283			
Hosmer and Lemeshow Test (Chi-square)	4.016			
Pr > Chi-square	0.856			

**Table 3:** Linear Model—Dependent Variable WTP for Cryopreservation (N = 499).

Variable	Beta	SE	*t*-Test	Pr > |t|	Tolerance
Intercept	15.98	0.87	2.51	0.03	-
Age	0.04	0.02	0.88	0.49	0.49
Education	−0.04	0.11	0.14	0.36	0.87
Personal monthly income	0.26	0.09	3.75	0.04	0.61
Degree of religious observance—Traditional (The term ‘traditional’ covers a wide range of ideologies and levels of observance and is based on self-definition)	−0.11	0.12	−2.81	0.03	0.91
Degree of religious observance—Religious (The term ‘religious’ refers to Jews who follow the traditional Jewish religion)	−0.17	0.22	−3.20	0.00	0.69
Family Status—Married	−0.08	0.05	1.77	0.12	0.54
Factor—Support	0.04	0.06	1.80	0.39	0.57
Factor—Continuation	0.07	0.05	1.03	0.12	0.97
Factor—Justification	0.06	0.10	0.03	0.16	0.70
Importance of the risk of infertility	0.22	0.10	0.21	0.00	0.63
Importance of Chance of pregnancy from frozen semen	0.04	0.09	−0.42	0.41	0.66
Importance of the option of sperm cryopreservation for chosen period of time	0.06	0.10	0.96	0.26	0.52
Initial registration fee to sperm bank and cryopreservation (One-time payment)	−0.21	0.10	−0.27	0.00	0.52
Annual fee for cryopreservation and storage	−0.02	0.07	2.51	0.70	0.77
F Value	9.17				
Pr > F	0.000				
R-squared	0.31				
Adj R-squared	0.29				

## Data Availability

The data presented in this study are available on request from the corresponding author. The data are not publicly available due to restrictions eg privacy.

## Appendix A

**Table A1:** The WTP Question.

The following question relates to a person’s desire to preserve genetic material—sperm so that it may be used during the person’s lifetime, after a serious illness and/or after death. People who are likely to consider sperm cryopreservation are mainly: soldiers (risk of death, physical injury, or illness during military service), patients with a serious disease (before chemotherapy for cancer or taking drugs that may affect the quality of sperm), men working in hazardous occupations (work in factories with hazardous chemicals or environmental pollution, x-rays or a nuclear reactor, or at a job with risk, such as firefighters, policemen, construction workers), and contact sports like football.
Over the past decade, awareness of the need for sperm cryopreservation has increased, due to a decline in sperm quality in Israel and abroad. See www.poriyut-guide.com. When answering the questionnaire, imagine that you must decide whether or not to cryopreserve sperm. What is the maximum amount, in $(Since the study was conducted in Israel, the monetary values were measured in Israeli New Shekels. I converted into dollars according to the dollar exchange rate on 23 February 2021, whereby, 1 ILS = 0.3063USD. The resulting dollar values were rounded according to mathematical rules for rounding numbers), that you are willing to pay the sperm bank annually for depositing your frozen sperm packets in the sperm bank for professional medical storage? a. $0–31 b. $31–92 c. $92–184 d. $ 184–276 e. $276–368 f. $368–460 g. $460–613 h. Above $613 Specify amount __________

**Table A2:** Attributes and levels included in the CA study.

Attribute	Levels and Definition
Risk of infertility (%)	Low (less than 20%); High (over 80%)
Chance of initiating a pregnancy from cryopreserved sperm (%)	10; 25; 30; 50
Option of sperm cryopreservation for chosen period of time (Years)	5; 10
Price of initial registration to sperm bank and cryopreservation One-time payment ($)	0; 92; 184; 306; 368
Annual fee for cryopreservation and storage ($) (must be paid every year)	46; 153

**Table A3:** Difference between choices in CA study.

Scenario	Your Chance of Infertility in the Future Difference between Option B and Option A (Option B—Option A)	Chance of Initiating a Pregnancy from Frozen Sperm Difference between Option B and Option A (Option B—Option A)	Option of Cryopreserving Sperm for Chosen Period Difference between Option B and Option A (Option B—Option A)	Price of Initial Registration to Sperm Bank and Cryopreservation (One-Time Payment) $ (Option B—Option A)	Annual Fee for Cryopreservation and Storage Difference between Option B and Option A $ (Option B—Option A)
Scenario 1	0	20%	5	0	−107
Scenario 2	60%	15%	0	0	−107
Scenario 3	60%	15%	0	122	0
Scenario 4	60%	0	5	184	0
Scenario 5	60%	20%	0	122	−107
Scenario 6	0	40%	5	122	−107
Scenario 7	0	15%	5	−184	0
Scenario 8	60%	0	0	−184	−107
Scenario 9	0	5%	0	−92	0
Scenario 10	60%	15%	0	−92	−107

**Table A4:** An example of one of the pairwise choices.

Option A	Option B	Case 10
Low (less than 20%)	High (more than 80%)	Risk of infertility
10%	25%	Chance of initiating a pregnancy from frozen sperm
5 years	5 years	Option of sperm cryopreservation for chosen period of time (Years)
$184	$92	Initial registration fee to sperm bank and cryopreservation (One-time payment)
$153	$46	Annual fee for cryopreservation and must be paid every year (storage)
Prefer option A	Prefer option B	Which option do you prefer?

**Table A5:** Importance degree of factors relevant to the decision to cryopreserve sperm.

Indicators	M	SD
The importance respondents attribute to the risk of being infertile	4.34	1.02
The importance respondents attribute to the chances of initiating a pregnancy from sperm that was cryopreserved	3.87	1.11
The importance respondents attribute to the possibility of cryopreserving sperm for a chosen period of time	3.55	1.17
The importance respondents attribute to the initial registration fee and providing semen to the laboratory for cryopreservation (one-time fee)	2.90	1.26
The importance respondents attribute to the annual price for cryopreservation and storage (must be paid annually)	3.19	1.20

**Table A6:** Zero-order Pearson correlation matrix among the attributes of the opinions pertaining to claims regarding freezing cryopreservation.

	1	2	3	4
The importance respondents attribute to the risk of being infertile	-			
The importance respondents attribute to the chances of initiating a pregnancy from sperm that was cryopreserved	0.502 **	-		
The importance respondents attribute to the possibility of cryopreserving sperm for a chosen period of time	0.425 **	0.503 **	-	
The importance respondents attribute to the initial registration fee and providing semen to the laboratory for cryopreservation (one-time fee)	−0.044	0.022	0.104 *	-
The importance respondents attribute to the annual price for cryopreservation and storage (must be paid annually)	−0.020	0.040	0.137 **	0.683 **

* significance at 5%; ** significance at 1%.

**Table A7:** The opinions pertaining to claims regarding freezing cryopreservation.

Item	N	SD	M	95% CI
Every soldier should freeze sperm before beginning his military service.	499	3.027	5.20	[4.93, 5.46]
Every man should deposit sperm in a sperm bank against the possibility that he might be diagnosed with cancer in the future.	499	2.905	5.23	[4.97, 5.48]
Every soldier setting out on a military action should deposit sperm in a sperm bank	499	2.924	5.06	[4.80, 5.31]
Parents who try to find a woman to bear a child from their son’s sperm do so because they want grandchildren.	499	2.947	5.92	[5.66, 6.17]
Soldiers don’t have to consider depositing sperm in a sperm bank.	499	3.027	5.09	[4.82, 5.35]
I would recommend to my son that he deposit sperm as a precaution before beginning military service.	499	2.834	4.23	[3.98, 4.47]
Every man should deposit sperm in a sperm bank only if and when he is diagnosed with cancer.	499	2.629	5.04	[4.80, 5.27]
Bereaved parents whose son died of cancer should try to find a woman to bear a child from their son’s sperm.	499	2.610	4.33	[4.10, 4.55]
Cryopreservation of combat soldiers’ sperm guarantees their genetic continuity if they fall in action.	499	2.753	5.68	[5.43, 5.92]
Cryopreservation of combat soldiers’ sperm guarantees their spiritual continuity if they fall in action.	499	2.893	4.33	[4.07, 4.58]
Bereaved parents who try to find a woman to bear a child from their son’s sperm are not considering the child’s welfare.	499	2.811	5.68	[5.43, 5.92]
Bereaved parents whose son fell in action should try to find a woman to bear a child from their son’s sperm.	499	3.027	5.20	[4.93, 5.46]
Sperm should be taken from a man after death only if he stated in his lifetime that he wishes this to be done in order to sire a child after his death.	499	2.905	5.23	[4.97, 5.48]

**Table A8:** Principal Component Analysis.

Items	Factor 1 (Support)	Factor 2 (Justification)	Factor 3 (Continuation)
1_Every soldier should freeze sperm before beginning his military service.	0.794		
2_Every man should deposit sperm in a sperm bank against the possibility that he might be diagnosed with cancer in the future.	0.748		
3_Every soldier setting out on a military action should deposit sperm in a sperm bank	0.674		
5_Soldiers don’t have to consider depositing sperm in a sperm bank.	0.587		
6_I would recommend to my son that he deposit sperm as a precaution before beginning military service.	0.758		
4_Parents who try to find a woman to bear a child from their son’s sperm do so because they want grandchildren.		0.732	
8_Bereaved parents whose son died of cancer should try to find a woman to bear a child from their son’s sperm.		0.596	
9_Cryopeservation of combat soldiers’ sperm guarantees their genetic continuity if they fall in action.		0.725	
10_Cryopreservation of combat soldiers’ sperm guarantees their spiritual continuity if they fall in action.		0.674	
12_Bereaved parents whose son fell in action should try to find a woman to bear a child from their son’s sperm.		0.658	
7_Every man should deposit sperm in a sperm bank only if and when he is diagnosed with cancer.			0.696
11_Bereaved parents who try to find a woman to bear a child from their son’s sperm are not considering the child’s welfare.			0.643
13_Sperm should be taken from a man after death only if he stated in his lifetime that he wishes this to be done in order to sire a child after his death.			0.634
Reliability (Cronbach’s Alpha Coefficient)	0.85	0.80	0.42
R-squared	27.09%	11.69%	22.25%
M	5.13	4.94	5.90
SD	2.33	2.07	1.88

**Table A9:** Zero-order Pearson correlation matrix among the factors of the opinions pertaining to claims regarding freezing cryopreservation.

	Factor 1 (Support)	Factor 2 (Justification)
Factor 1 (Support)	-	
Factor 2 (Justification)	0.653 **	-
Factor 3 (Continuation)	−0.044	0.014

** significance at 1%.
